# Alkaline phosphatase in nasal secretion of cattle: biochemical and molecular characterisation

**DOI:** 10.1186/s12917-014-0204-9

**Published:** 2014-09-05

**Authors:** M Faizal Ghazali, HH Caline Koh-Tan, Mark McLaughlin, Paul Montague, Nicholas N Jonsson, P David Eckersall

**Affiliations:** Institute of Biodiversity, Animal Health and Comparative Medicine, University of Glasgow, Bearsden Rd, Glasgow, G61 1QH UK; School of Veterinary Medicine, University of Glasgow, Bearsden Rd, Glasgow, G61 1QH UK; Institute of Infection, Immunity and Inflammation, University of Glasgow, Bearsden Rd, Glasgow, G61 1QH UK

**Keywords:** Bovine, Nasal secretion, Alkaline phosphatase, Isoelectric focusing, RT-PCR

## Abstract

**Background:**

Nasal secretion (NS) was investigated as a source of information regarding the mucosal and systemic immune status of cattle challenged by respiratory disease. A method for the collection of substantial volumes (~12 ml) of NS from cattle was developed to establish a reference range of analytes that are present in the NS of healthy cattle. Biochemical profiles of NS from a group of 38 healthy Holstein-Friesian cows revealed high alkaline phosphatase (AP) activity of up to 2392 IU/L. The character and source of the high activity of AP in bovine NS was investigated.

**Results:**

Histochemical analysis confirmed the localization of the AP enzyme activity to epithelial cells and serous glands of the nasal respiratory mucosa. Analysis of mRNA levels from nasal mucosa by end point RT-PCR and PCR product sequencing confirmed that the AP was locally produced and is identical at the nucleotide level to the non-specific AP splice variant found in bovine liver, bone and kidney. Analysis by isoelectric focussing confirmed that AP was produced locally at a high level in nasal epithelium demonstrating that AP from nasal secretion and nasal mucosa had similar pI bands, though differing from those of the liver, kidney, bone and intestine, suggesting different post-translational modification (PTM) of AP in these tissues.

**Conclusions:**

A nasal isozyme of AP has been identified that is present at a high activity in NS, resulting from local production and showing distinctive PTM and may be active in NS as an anti-endotoxin mediator.

## Background

Bovine respiratory disease (BRD) is an important economic challenge to beef production globally and is estimated to cost the UK cattle industry £60 million annually [1]. We wished to investigate bovine nasal secretion (NS) as a source of information regarding the mucosal and systemic immune status of cattle in health and in relation to BRD.

To use a body fluid such as NS for detection and monitoring of disease it was necessary to establish a reference range of analytes that are present in the NS of healthy cattle. However, for bovine NS there is no information on the composition of the secretion, possibly due to the perceived difficulty of collecting a sufficient volume of NS for analysis. A method was therefore developed which allowed collection of over 10 ml of NS, sufficient for multiple investigations. Thereafter in the course of biochemical analysis of NS, using a panel of tests that are used in veterinary clinical biochemistry analysis, it was found that the activity of the enzyme, alkaline phosphatase (AP; EC 3.1.3.1) in the NS was markedly higher (up to 2392 IU/L) than the activity found in healthy bovine serum (20–80 IU/L).

In cattle, as in other animals, AP exists as several isozymes in different tissues, encoded by two known genes: one for the intestinal form (IAP) and the other for the non-specific AP which occurs in bone (BAP), liver (LAP) and placenta (PLAAP) [2–4]. A major biochemical function of the enzyme family is to dephosphorylate molecules with consequent change in function of those molecules. Recent investigation into the function of AP has revealed actions that could be relevant to its presence in NS. One of these is the dephosphorylation of bacterial lipopolysaccharides (LPS) resulting in detoxification of the LPS [5–8]. For IAP this has been shown to have a role in the maintenance of gut homeostasis, favouring the proliferation of commensal bacteria over pathogens such as *Salmonella typhimurium* [9]. This activity is proposed to be related to local immunomodulating effects, likely via regulation of the LPS-toll-like receptor 4 (TLR4) interactions between gut microflora and intestinal epithelium [10].

Within the respiratory tract, AP activity has been previously demonstrated within the cell and on the surface of respiratory epithelium in humans, where it is able to dephosphorylate ATP to AMP and to adenosine, important for mucociliary clearance [11]. The enzyme was also reported several decades ago in human nasal mucosa [12] and more recently described in this tissue and, though in micro litre volumes, of NS of guinea pigs [13]. Alkaline phosphatase activity has also been found in the olfactory epithelium of mice and rats [14]. Apart from these reports, the activity and production of AP in NS and nasal mucosa has not, to our knowledge, been documented. The ability to collect substantial (ml) volumes of bovine NS has enabled a biochemical analysis of the NS and investigation of the possibility that the high activity of AP in NS has a significant biological function.

The AP in NS could originate from either local synthesis or secretion from cells in the bovine nasal epithelium or it could be a result of leakage from serum. However the latter theory is not supported by the finding of higher activity of AP in NS than in serum unless a mechanism to export the AP against a concentration gradient is present in this tissue. The objective of this investigation was to characterise and identify the source of the AP in bovine NS and establish if this AP has similarity to established isoforms of the enzyme.

## Methods

### Animals and collection of nasal secretion

**Figure 1:**
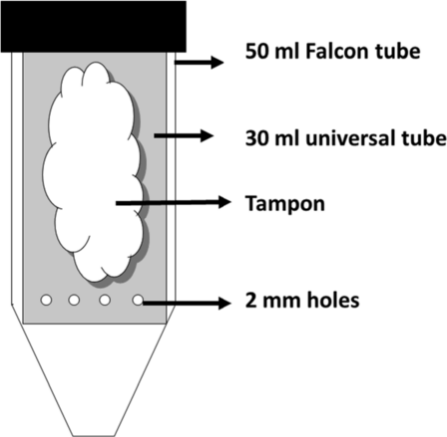
**Schematic representation of a collecting tube for nasal secretion.** The saturated tampon from nostril was inserted into a modified collecting tube consisted of a 30 ml universal tube with four × 2 mm holes drilled into the bottom, inserted in a 50 ml Falcon tube.

38 Holstein-Friesian cows aged 2–5 years were sampled from a herd of 90 lactating cows from University of Glasgow Cochno Farm. The animal experiments were carried out with the approval of the University of Glasgow MVLS College Ethics Committee and complied fully with the Home Office of Great Britain and Northern Ireland “Animals (Scientific Procedures) Act 1986”. Clinical examination was performed to ensure the cows were clinically healthy and free from respiratory disease. The cows were restrained in a cattle crush during the procedure. A commercially available tampon was inserted into one nostril and slid gently in an upwards and backwards direction about 5–8 cm deep and then left in place for up to 15 minutes [15]. The tampon was then removed from the nostril by gently pulling on the attached string and weighed, as a measure of the NS uptake before being inserted into a modified collecting tube (Figure 1). The cows were observed during collection to ensure that there were no signs of discomfort but no signs of discomfort were observed at any time in any cow in the study. The modified tubes were centrifuged at 700 g for 10 minutes at 4°C in a procedure similar to that of Lu and Esch [16]. Nasal secretion collected at the bottom of Falcon tube was transferred into 1.5 ml tubes and stored at −80°C until further analysis.

### Protein extraction for biochemical and immunological analysis

Nasal mucosa, small intestine, heart, liver and kidney samples from 6 adult cattle were collected at a slaughterhouse and processed within 24–48 hours. Nasal mucosa was gently cut and separated from the nasal conchae using a forceps and blade. Intestinal mucosa was obtained by gently scraping the mucosa of the small intestine with a metal spatula. Tissues were snap-frozen in liquid nitrogen and stored at −80°C prior to extraction. Two grams from each tissue were washed with isotonic saline solution and homogenized (Janke & Kunkel Ultra Turrax T25) in 5 ml saline. AP was extracted from 2 samples of crushed cancellous bone obtained from the metacarpal bone of 2 cattle.

Tissue AP extracts were further processed by incubation with 100% n-butanol in ratio of 1:4 of n-butanol:protein homogenates, with gentle agitation at 4°C for 30 minutes [17]. Extraction with n-butanol facilitates the release of AP from membranes where it is anchored to phosphatidylinositol [18]. The samples were centrifuged at 1,500 g for 30 minutes at 4°C, the aqueous layer removed and centrifuged at 15,000 g for 90 minutes with the supernatant being the AP extract. The AP activity and the protein concentration in the AP extracts were determined as described below. The AP activity of tissue extracts was recorded as enzyme activity per gram of tissue extracted (U/g). The relative AP activity in the different extracts was compared by a Kruskal-Wallis test (GraphPad Prism 5.0, California, USA) with a significance level (α) set at 0.05.

### Biochemical and immunological analysis

The nasal secretions and tissue extracts were examined for biochemical composition using an Olympus A640 analyser operated by Veterinary Diagnostic Services, University of Glasgow. Enzymatic test were carried out at 37°C where measurement of AP were based on the recommendations of the German Society for Clinical Chemistry (GSCC) at a pH of 10.4. Analysis method of γ − glutamyl transferase (GGT) and aspartate transaminase (AST) were based on the recommendation of the International Federation of Clinical Chemistry (IFCC). Albumin was measured by a bromcresol green (BCG) binding assay, total protein by reaction with biuret, urea by reaction with 2-oxogluturate and nicotinamide adenine dinucleotide (NADH), creatinine by reaction with picric acid, total bilirubin by reaction with 3,5-dichlorophenyldiazonium tetrafluoroborate (DPD), calcium by reaction with o-Cresolphthalein-complexone (oCPC) and phosphate by reaction with molybdate. Measurements of electrolytes were carried out by ion-specific electrodes using an indirect potentiometry method. Measurement of bovine IgA and IgG concentrations using species-specific ELISAs (Bethyl Labs, Texas, USA) according to the manufacturer’s instructions.

### Histochemical analysis

Bovine nasal mucosa were collected for histology examination as described above with a 1 cm square dissected out and snap frozen in liquid nitrogen. Cryosections of 8 μm were fixed with acetone, stained with Vector® Red Alkaline Phosphatase Substrate Kit (Vector Labs, Peterborough, UK), in the presence or absence of levamisole inhibitor (Sigma-Chem Co, Poole, UK) counterstained with Gills Haematoxylin (Sigma-Chem Co, Poole, UK) and mounted with Histomount (National Diagnostics, New Jersey, USA). Images were taken with Olympus BX51 and attached DP71 camera (Olympus, Japan), using Cell^D software (Olympus, Japan).

### Total cellular RNA extraction

Tissue samples of 100 mg from the same cattle used for the preparation of tissue extracts were used for RNA preparation using PureZOL™ (Bio-Rad Laboratories, Hemel Hempstead, UK) according to the manufacturer’s protocol.

### End point reverse transcriptase – polymerase chain reaction (RT-PCR)

Reverse transcription (RT) reactions were performed on RNA samples from nasal mucosa, liver and small intestine. 2 μg RNA samples were denatured at 65°C for 5 minutes. 50 μl reactions comprising 2 μg of total RNA sample, 1 × First strand buffer, 0.5 mM each of dNTPs, 10 mM dithiothreitol, 3 μg random primers, 20 U of RNAseOut and 400 U M-MVL reverse transcriptase (Life Technologies Ltd, Paisley, UK) were incubated for 30 minutes at 37°C, 60 minutes at 42°C, 15 minutes at 70°C and quenched at 4°C. The PCR products were analysed on 2% (w/v) agarose gels containing ethidium bromide (0.5 μg/ml).

Primers were designed to amplify specific regions of the bovine intestinal AP (IAP) gene ALPI (NM_173987) and non-specific AP gene ALPL (NM_176858), respectively using an interactive web-based primer program algorithm, GeneFisher software version 1.2.2 (BiBiServe, Bielefeld, Germany), with a predicted size PCR product of 500 bp. The primer sequences are depicted in Table 1. A total volume of 25 μl PCR reaction was prepared, comprising 12.5 μl RedTaq® DNA polymerase buffer (Sigma Chem. Co, Poole, UK), 0.5 μl of each primer (5 pmol per reaction tube), and 200 ng gDNA. Amplification conditions were 30 cycles (94°C for five minutes, 94°C for one minute, 58°C for one minute, 72°C for one minute, 72°C for 10 minutes). The PCR products (2 μl) were visualised using ethidium bromide (0.5 μg/ml) stained agarose gel and quantified against a 100 bp mass ladder for DNA (Life Technologies, Paisley, UK).

**Table 1:** **Primer sequences used for RT**-**PCR**

**Gene**	**Accession number**	**Primer notation**	**Sequence 5** **′** **-** **3** **′**
*ALPI*	NM_173987	ALPI-F	GGGAGTGGTGACCACCTCCA
		ALPI-R	GTCAATGCGGCCTCCCTCCA
*ALPL*	NM_176858	ALPL-F	GACAGCTGCCCGCATCCTCA
		ALPL-R	CCTTCTCATCCAGCTCATACTCCA

### Sequencing

PCR products from RT-PCR reactions were purified using a QIAquick PCR purification kit (Qiagen, Crawley, UK) according to the manufacturer’s instructions. The PCR products were sequenced using Big Dye v3.1 Terminator (Life Technologies, Paisley, UK) in 10 μl reaction volume containing 200–500 ng template, 1X sequencing buffer, 32 mM primer and 1x Big Dye mix. The sequencing reactions were carried out in two random nasal mucosa samples with forward and reverse primers in separate reactions. The sequencing products were ethanol precipitated, resuspended in formamide and read using capillary electrophoresis on Applied Biosystems 3130XL Genetic Analyzer (Hitachi, Tokyo, Japan).

### Isoelectric focusing (IEF)

The pI of the various AP isozymes was determined by separation using Invitrogen Novex® pH 3–7 IEF Gel (Life Technologies, Paisley, UK). Samples of bovine NS and tissue extracts from NS, nasal mucosa, small intestine, liver, kidney and bone were selected at random from the available samples for the separation of AP isozymes. Samples were diluted to an AP activity of 300–350 IU/L in IEF sample buffer pH 3–7 (Life Technologies, Paisley, UK) prepared according to manufacturer’s instructions. Ten microliters of the prepared sample were loaded into each well such that equal activities of AP were loaded into each well. Isoelectric focussing was conducted at 4°C using the following voltage gradient: 100 V, 1 hour; 200 V, 1 hour; 500 V, 30 minutes. The gel was then stained with Pierce 1-Step™ NBT-BCIP ALP substrate solutions at pH 9.2 (Thermo Fisher Scientific Inc, Illinois, USA) for 5 hours at 37°C. The pI of focussed bands was estimated in comparison to pH 3–10 standards (Serva Electrophoresis, Heidelberg, Germany) which were run in a track of the IEF gels, with the track being excised and stained separately with Coomassie blue following isoelectric focusing.

## Results

### Biochemistry of bovine nasal secretion

A volume of between 5–12 ml of NS was collected from each of 38 Holstein-Friesian dairy cows. Protein concentrations in nasal secretion ranged from 9 to 34 g/L. Following biochemical analysis the mean (±SD) AP activity in NS was 1239 ± 553 IU/L and was up to 16-fold higher than the serum reference range for AP (Table 2) while GGT activity was 2.6-fold higher than the bovine serum GGT reference range. Concentrations of total protein, albumin, calcium, phosphate, sodium and chloride were lower than the serum reference range while urea, creatinine, bilirubin and aspartate transaminase levels were comparable to serum reference ranges. The concentrations of IgA and IgG were 0.5 - 2 g/L and 0.2 - 1.9 g/L respectively.

**Table 2:** **Biochemical and immunological compositions of bovine nasal secretion** (**n** = **38**)

**Analytes** **(unit)**	**Median** ** (Range)**	**Reference range** ** (Bovine serum)** ^**a**^
Urea (mmol/L)	3.1 (1.8 - 6.1)	0 - 8
Calcium (mmol/L)	1.1 (0.6 - 1.4)	2.2 - 3.3
Phosphate (mmol/L)	0.8 (0.4 - 1.6)	1.1 - 2.8
Creatinine (μmol/L)	30 (5 - 61)	53 - 132
Total bilirubin (μmol/L)	0 (0 - 2)	0 - 8
Total protein (g/L)	14 (9 - 34)	52 - 84
Albumin (g/L)	3 (0 - 5)	21 - 34
Sodium (mmol/L)	60 (18 - 98)	135 - 151
Potassium (mmol/L)	2.4 (0.6 - 3.9)	3.2 - 5.8
Chloride (mmol/L)	19 (14 - 34)	96 - 111
Alkaline phosphatase (IU/L)	1155 (144 - 2392)	20 - 80
Aspartate transaminase (IU/L)	97 (50 - 184)	10 - 140
Gamma-glutamyltransferase (IU/L)	68 (28 - 109)	0 - 27
IgA (g/L)	1.2 (0.5 - 2.0)	1.8 - 3.9^b^
IgG (g/L)	0.5 (0.2 - 1.9)	0.1 - 0.7^c^

^a^Laboratory reference range for bovine serum; ^b^Morgan et al., [19]; ^c^Duncan et al., [20].

### Distribution and localisation of AP activity

The extracts from nasal mucosal tissue (n = 6) had mean AP activities which were not significantly different from the mean activity extracted from other bovine tissues known to produce this enzyme: liver, intestine and kidney. All of these extracts had significantly higher AP activities per gram of wet tissue extracted compared with the heart tissue (Table 3) that served as a negative control.

**Table 3:** **AP activity in bovine tissue extracts** (**n** = **6**)

**Tissue**	**Median** ** (IU/** **g) (** **Range)**
Nasal mucosa	14.3 (1.0 - 27.6)
Intestinal mucosa	6.5 (2.2 - 24.0)
Heart	0.6 (0.2 - 1.1)*
Kidney	7.4 (3.9 - 16.2)
Liver	8.3 (1.0 - 27.2)

*AP activity was significantly different from all other tissue extracts (P < 0.05).

**Figure 2:**
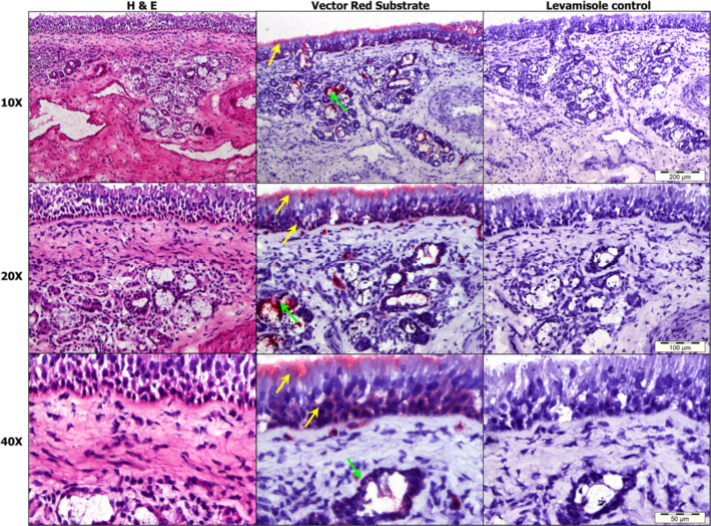
**Histology and alkaline phosphatase activity histochemistry of nasal mucosa.** Routine staining of nasal mucosa with Haematoxilin and Eosin (left column) and histochemical staining of AP activity using Vector Red substrate (central column) showed strong AP activity in the mucus at the luminal surface and in the basal cell layers of the epithelium (yellow arrow); as well as in serous glands (green arrow). Levamisole, a non-specific AP inhibitor, abolished the histochemical staining of these cells (right column). Image captures at x10, x20 and x40 magnifications are shown with appropriate scale bars.

Histochemistry using an AP activity stain showed strong AP activity (Figure 2) in the luminal and basal ends of nasal epithelium (yellow arrow) and serous glands (green arrow). AP activity staining was abolished in the presence of levamisole a known non-specific AP inhibitor.

### Analysis of nasal mucosa AP mRNA abundance levels

**Figure 3:**
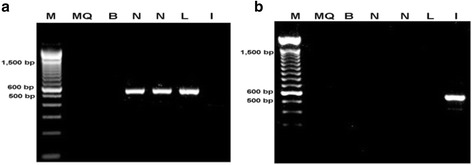
**Alkaline phosphatase mRNA analysis from bovine tissues by RT-** **PCR.** Bovine tissues examined by RT-PCR to determine expression of genes for AP. The presence of a single 500 bp DNA fragment corresponding to the predicted product size was visualised when using **(a)** non-specific AP primers and **(b)** intestinal AP primers. PCR products were present in nasal mucosa only when non-specific AP primers were used. M = Marker (100 bp DNA ladder); MQ = high purity water; B = blank; N = nasal mucosa; L = liver; I = intestine.

A single PCR product at the predicted size of 500 bp was detected in the nasal mucosa using primers for bovine non-specific AP gene ALPL (NM_176858) (Figure 3a) but not with primers for the intestinal AP (IAP) gene ALPI (NM_173987) (Figure 3b). Liver and intestine cDNAs served as positive and negative controls for each primer pair. Sequencing of PCR products from two biological samples of nasal tissue showed homology to the ALPL splice variant.

### Isoelectric focusing of AP isoforms

**Figure 4:**
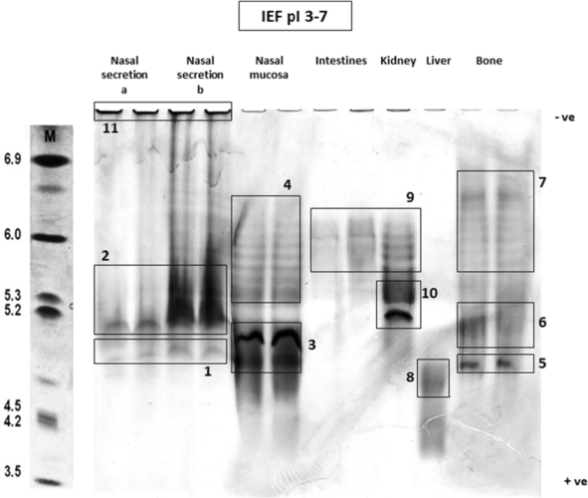
**Isoelectric focussing of alkaline phosphatase in bovine tissue and nasal secretion.** Bovine tissue extracts and nasal secretion separated on isoelectric focusing gels over a pH 3–7 range and stained with 5-Bromo-4-chloro-3-indolyl phosphate with nitro blue tetrazolium chloride (BCIP/NBT) substrate. M = IEF standard protein marker (stained with Coomassie blue); −ve = cathode; +ve = anode. Two different samples of NS were run in duplicate. Samples of extracts from nasal mucosa, bone and intestine were run in duplicate with liver and kidney extract being run in single tracks. Annotated boxes around isoforms bands as discussed in the text.

The AP from various bovine tissues separated by IEF and stained by zymography with an AP susbstrate, are shown in Figure 4 including boxes around selected results. The IEF demonstrated that AP from the NS (run in duplicate) had isoforms forming bands of AP activity with pI of 4.8 (Figure 4–1) and 5.0-5.3 (Figure 4–2). There were inter-individual difference found in the AP bands from NS samples at pI 5.0-5.3 illustrated by NS samples (a) and (b) with sample (b) showing greater activity and had a smeared appearance. The nasal mucosal extract also had major bands with pI 4.8-5.2 (Figure 4–3) and several minor bands with pI of 5.3 - 6.2 (Figure 4–4). Bovine bone AP had an isoform with pI of 4.8 (Figure 4–5) and 5.0-5.3 (Figure 4–6), with several bands with pI of 6.0 – 6.7 (Figure 4–7). The liver AP focussed with lower pI showing a major band at pI 4.6 (Figure 4–8). Intestinal and kidney AP had isoforms with pI of 5.4-6.2 (Figure 4–9) while kidney AP also having major bands between with pI of 5.2 and 5.3 (Figure 4–10). The pI of the isoforms were estimated by comparison to standard protein of known pI value run on the same IEF gel with the track of the standard proteins separated and visualised with Coomassie blue protein stain rather than with AP zymography. Although diluted to a similar AP activity, notable differences were observed in the band intensity of the AP isoforms. Close inspection of the loading well at the cathodal end of the gel, for the NS samples showed a deposit of AP activity, suggesting that a proportion of the AP in these samples were unable to enter the polyacrylamide gel, possibly due to the formation of large aggregates.

## Discussion

This investigation had the original objective to collect and characterise bovine NS as a readily available biological fluid in the search for biomarkers of BRD. However prior to this, it was necessary to establish the normal components of the fluid and their concentration in NS from healthy cattle. Swabbing the nasal tract proved to be an inefficient method of collection of the fluid. A system was therefore developed which allowed bovine NS to be collected in sufficient volume for analysis. Previous studies have collected NS on tampons [15] but in this study the sample was removed from the absorbent by centrifugation using modified centrifuge and universal tubes, which allowed up to 12 ml of NS to be collected from each animal.

Biochemical analysis showed that most analytes were at similar or lower concentrations to those in bovine serum. Unexpectedly, it was found that the activities of AP and GGT were greater by factors of close to ten-fold and three-fold respectively in the NS than in serum from healthy animals. To our knowledge there are no previous reports of the activities of these enzymes in NS being greater than that found in serum or plasma. Indeed in the classic text on AP by McComb et al. [2] there is no mention of AP in nasal mucosa or NS and only one reference to AP in olfactory cells related to the nasal cavity [21]. The lack of prior reports on the activity of AP in NS is likely to be due to the difficulty of collection of sufficient volumes of this fluid from animals such as laboratory rodents and reluctance of human volunteers to undertake NS collection. Whether the high activity of GGT in NS is also produced locally is an open to question and would require confirmation by gene expression or by histochemical analysis.

Alkaline phosphatase is synthesised in several tissues in mammals and distinct gene products have been identified in cattle with one form being produced in the intestine (IAP) and a second isoform, non-specific AP, being synthesised in liver, kidney and bone [3,22]. Recently, a study conducted by Yang et al. [4] discussed the evolution of AP genes, indicating that mammals have lost 1 of 3 clades present in zebrafish, however they also indicated that IAP is regulated by Myd88 dependent innate immune signalling with activity in reducing host responses to inflammatory microbiota products [4]. An activity based AP specific histochemical stain was used on sections of bovine nasal epithelia and indicated that AP is present in the mucus layer and in secretory cells of the nasal epithelium. Using a similar histochemical approach, the enzyme has been previously identified in human nasal mucosa [12] in the nasal epithelium of guinea pigs [13] and the olfactory epithelium of mice and rats [14] but only limited volumes of NS were available for analysis. The study of AP in the nasal epithelium of guinea pigs [13] did detect AP activity in NS collected by lavage, but only a total volume of 30 μl of lavage fluid was collected per guinea pig, which would not be a sufficient volume for many investigations. Measuring AP activity from nasal mucosa for comparison to extracts of other bovine tissues revealed that the activity of AP per gram of tissue from nasal mucosa was equivalent to that of liver, kidney and intestine indicating that nasal mucosa is a major site for AP synthesis and secretion.

Enzyme isoforms can be separated by electrophoretic means to aid in identification of relative isoforms. Isoelectric focusing was able to separate the AP and allowed the enzyme activity to be detected by AP activity staining. Although similar AP activity was loaded on to the IEF gel, inspection revealed notable differences in the band intensities. This difference was possibly due to differences in the ability of the AP enzyme to penetrate the polyacrylamide gel which was especially evident in the NS samples (Figure 4–11). This could be due to the formation of aggregates, possibly with high molecular weight and complex proteoglycans contained in nasal mucus remaining at the sample well. Smearing of IEF track from NS sample (b) (Figure 4–2) and its greater activity compared to sample (a) may be due to partial degradation of AP molecules during sample preparation which could allow greater penetration of the gel by smaller AP forms. Staining was carried out at pH 9.2 and as there is a possibility that different AP isoform would optimally stain at different pHs and this may also contribute to the variation seen in AP activity on the IEF gel. Nevertheless, the AP isoforms in NS had pIs in the same region as those of the nasal mucosa extracts (pH 4.2-6.8) but were clearly different from the extracts of other tissues. The differences in IEF migration of the AP extracts is likely to be due to post translational modifications (PTMs) such as glycosylation or phosphorylation, the elucidation of which would be the subject of further investigation.

PCR using primers based on the published sequences of bovine intestinal [4] and non-specific AP [3] established that the nasal mucosa PCR product is identical to the bovine non intestinal AP messenger and was not related to intestinal AP. This was further confirmed by sequencing the PCR product which showed complete sequence identity with the published bovine non-specific AP sequence [22].

Recent studies of the biological function of AP have revealed that the enzyme has the ability to dephosphorylate lipopolysaccharide (LPS) endotoxin from Gram negative bacteria [23–25] reducing the toxic effects of LPS [26] and, in the intestine, can be induced by Resolvin E1, an anti-inflammatory derivative of omega-3 fatty acids [27]. Thus intestinal AP has a major function in reducing the effects of LPS from intestinal bacterial flora [5] and may have a similar function in livestock [28]. If AP in NS has the same activity on LPS then its presence in this secretion could contribute to the host defences by acting against invading pathogens in the respiratory tract responsible for BRD. It is of interest that AP in bronchial and alveolar fluid is believed to function in the extracellular dephosphorylation of ATP to fuel the activity of cilia in the airways [11] while there may also be interaction between ATP and nasal AP in minimizing inflammation as has been suggested for IAP [8]. This may be relevant to its presence in NS, while de-phosphorylation of the proinflammatory nucleotide uridine diphosphate (UDP), an action of IAP [29] and the relative stability of UDP on the airway surface of human [30] could provide nasal AP as a potential anti-inflammatory action with therapeutic value for the treatment of airway diseases. Furthermore in this species, NS can be transferred to the mouth as cattle have the tendency to lick their muzzle and nostrils so that the nasal AP could be effective in the rumen as an anti-endotoxin with a de-phosphorylation activity [8].

## Conclusion

This investigation has shown that AP is present in substantial activity in bovine NS. We have demonstrated that AP activity found in bovine NS is produced locally in the nasal epithelium and does not result from transfer from the serum. This is based on the findings that AP was extracted from nasal epithelium with a similar efficiency as from liver, bone, kidney or intestinal tissue, AP activity was detected by histochemistry in sections of the nasal epithelium and the mRNA for bovine non-specific AP was present in this tissue. Isoelectric focusing demonstrated differences between the AP of NS and extracts of AP from other tissues with differences likely to be resulting from PTMs. The discovery of AP in nasal epithelium and NS in cattle presents opportunities for further investigation of the role of the enzyme in the susceptibility of mammals to infection through the nasal route.
